# Fibroblast Growth Factor-1 Activates Neurons in the Arcuate Nucleus and Dorsal Vagal Complex

**DOI:** 10.3389/fendo.2021.772909

**Published:** 2021-12-20

**Authors:** Brandon L. Roberts, Eric J. Kim, Sarah R. Lindsley, Katherine G. Tennant, Paul Kievit

**Affiliations:** Division of Cardiometabolic Health, Oregon National Primate Research Center, Beaverton, OR, United States

**Keywords:** fibroblast growth factor-1 (FGF1), proopiomelanocortin (POMC), neuropeptide Y (NPY), arcuate nucleus of the hypothalamus (ARH), dorsal vagal complex (DVC), diabetes

## Abstract

Central administration of fibroblast growth factor-1 (FGF1) results in long-lasting resolution of hyperglycemia in various rodent models, but the pre- and postsynaptic mechanisms mediating the central effects of FGF1 are unknown. Here we utilize electrophysiology recordings from neuronal populations in the arcuate nucleus of the hypothalamus (ARH), nucleus of the solitary tract (NTS), and area postrema (AP) to investigate the mechanisms underlying FGF1 actions. While FGF1 did not alter membrane potential in ARH-NPY-GFP neurons, it reversibly depolarized 83% of ARH-POMC-EGFP neurons and decreased the frequency of inhibitory inputs onto ARH-POMC-EGFP neurons. This depolarizing effect persisted in the presence of FGF receptor (R) blocker FIIN1, but was blocked by pretreatment with the voltage-gated sodium channel (VGSC) blocker tetrodotoxin (TTX). Non-FGF1 subfamilies can activate vascular endothelial growth factor receptors (VEGFR). Surprisingly, the VEGFR inhibitors axitinib and BMS605541 blocked FGF1 effects on ARH-POMC-EGFP neurons. We also demonstrate that FGF1 induces c-Fos in the dorsal vagal complex, activates NTS-NPY-GFP neurons through a FGFR mediated pathway, and requires VGSCs to activate AP neurons. We conclude that FGF1 acts in multiple brain regions independent of FGFRs. These studies present anatomical and mechanistic pathways for the future investigation of the pharmacological and physiological role of FGF1 in metabolic processes.

## Introduction

Fibroblast growth factor -1 (FGF1) is part of the larger FGF family and functions as a growth factor and signaling protein (1). FGF1 is involved in multiple biological processes, including cell proliferation of preadipocytes and neural stem cells, differentiation of neuroepithelial cells, and tumor progression (1–4). More recently, FGF1 has been implicated in the regulation of glucose homeostasis and proposed as a potential therapeutic for type 2 diabetes (5–9). A single intracerebroventricular (i.c.v.) injection of FGF1 into the lateral ventricle of diabetic rodent models, such as *db/db* or *ob/ob* mice and Zucker diabetic fatty rats (ZDF), induces a short-term reduction in food intake and chronic reduction in hyperglycemia, lasting up to 18 weeks in leptin-deficient *ob/ob* mice (6–8). While the mechanism by which FGF1 induces the remission of hyperglycemia is unknown, there is sufficient evidence to suggest that its actions involve interplay between the brain and periphery, leading to long-term changes in hepatic and pancreatic function (6).

Previous studies demonstrated that direct injection of FGF1 into the ARH is sufficient to reduce hyperglycemia in rodents; however, whether FGF1 acts directly on ARH neurons, which subpopulation of neurons respond to FGF1, and what receptor(s) mediate the effects of FGF1, is unknown (7). Two neuronal phenotypes in the ARH that are involved in the central regulation of glucose and overall energy homeostasis are neuropeptide Y (NPY) and proopiomelanocortin (POMC) neurons (10–12). The activity of ARH-POMC neurons is mediated through several signals involved in energy balance and glucose homeostasis. They are inhibited by orexigenic signals including ghrelin, low glucose and PYY_3-36_, and are directly activated by anorexic signals, including insulin, high glucose, and leptin (13–16). The activity of ARH-POMC neurons is partly regulated by inhibitory inputs from the orexigenic ARH-NPY neurons (16, 17). ARH-NPY neurons co-express agouti-related peptide (AgRP), are GABAergic, and directly synapse onto ARH-POMC neurons to modulate food intake and energy homeostasis (10–12, 18). Additionally, they can induce food intake by inhibiting melanocortin receptor 4 (MC4R) containing neurons in the paraventricular nucleus of the hypothalamus (PVH) (19).

Interestingly, recent work demonstrates that the melanocortin system is required for the sustained effects of FGF1 on diabetes remission as Mc4r knockout (Mc4r^-/-^) mice or pretreatment with the MC4R antagonist SHU9119 blunts the long-term, but not acute, effects of FGF1 (20). The mechanisms underlying the acute effects of FGF1 remain unknown. While the role of the hypothalamus in mediating FGF1 effects are under current investigation, a key brain region involved in relaying autonomic signals between the brain and periphery that mediates acute changes in food intake is the dorsal vagal complex (DVC). The DVC contains glucose responsive neurons and has reciprocal connections with the gastrointestinal tract, liver and pancreas *via* efferent and afferent vagal fibers (21–24). It is comprised of the dorsal motor nucleus of the vagus (DMV), nucleus of the solitary tract (NTS), and area postrema (AP). The NTS receives direct inputs from the gut through vagal afferent fibers and relays this information throughout the brain (25). The AP is a circumventricular organ (CVO) vascularized by fenestrated capillaries, contains glucose sensitive neurons, and is a key player in multiple autonomic functions, including feeding and metabolism (26, 27). Together, the DVC is required for glucoprivic feeding and hepatic glucose production induced by nutrient sensing in the hypothalamus (23, 28). Central FGF1 injections preserve β-cell function and increase hepatic glucokinase activity, both of which may underlie the remission of hyperglycemia in diabetic rodent models (6, 8); however, whether the DVC is involved in the metabolic actions of FGF1 or if neurons within this region respond to FGF1 is unknown (21–24).

Here we determine whether FGF1 has direct actions on NPY or POMC neurons in the ARH and NTS, as well as unidentified AP neurons. Next, we address whether i.c.v. injections of FGF1 activate neurons in the DVC. Lastly, we elucidate a potential mechanism by which FGF1 acts on these neuronal subpopulations.

## Materials and Methods

### Animals

All animal procedures and experiments were approved by the Oregon National Primate Research Center Animal Institutional Care and Use Committee in accordance with the U.S. Public Health Service Policy on Humane Care and Use of Laboratory Animals and the National Institutes of Health *Guide for the Care and Use of Laboratory Animals*. Male POMC-EGFP (JAX stock #009593, The Jackson Laboratory) and NPY-GFP (JAX stock #006417, The Jackson Laboratory) transgenic mice on a C57BL/6 background were bred in-house (18, 29). All mice were group-housed at 25°C, under a 12/12-hr light/dark cycle, with food and water available *ad libitum*. For induction of DIO, mice were placed on a 60% high-fat diet (HFD) for 12-16 weeks (D12492, Research Diets). Mice ages 10-24 weeks were used for these studies. For electrophysiology studies mice were anesthetized in a chamber with isoflurane before sacrifice by decapitation.

### Brain Slice Electrophysiology

Brain slices were collected as previously described (30). The forebrain and brainstem were mounted adjacent to each other and sectioned simultaneously with a sapphire knife (Delaware Diamond Knives) yielding roughly three slices from each region (250-μm) per mouse. The experiment performed on each slice was randomized to obtain proper animal numbers while minimizing animal use. Slices were maintained at room temperature in a recording aCSF solution composed of (mM): 124 NaCl, 5 KCl, 2.6 NaH_2_PO_4_, 10 HEPES, 26 NaHCO_3_, 2 CaCl_2_, MgSO_4_, 5 dextrose, and bubbled using 95% 0_2_/5% C0_2_. For recordings, brain slices were transferred to a perfusion chamber containing aCSF maintained at 34-37°C. Neurons were visualized using an upright microscope (Zeiss Axoskop 2). Recording electrodes were back-filled with experiment-specific internal solutions as follows (mM): Current-clamp; 125 K-gluconate, 2 KCl, 5 HEPES, 10 EGTA, 5 MgATP (1 MgATP for ARH-NPY recordings), and 0.25 NaGTP. Voltage-clamp IPSC; 140 CsCl, 5 MgCl_2_, 1 BAPTA, 10 HEPES, 5 MgATP, 0.25 and NaGTP. All internal solutions were brought to pH 7.3 using KOH (voltage-clamp) or CsOH (IPSC) at 301-304 mOsm. IPSCs were recorded in the presence the competitive AMPA/kainate receptor antagonist CNQX (10 μM) and the selective NMDA receptor antagonist APV (50 μM) as previously described (30). Patch electrodes with a resistance of 3-5MΩ were guided to neurons with a motorized micromanipulator (MX7600 with MC1000e controller, SD Instruments). Patch-clamp recordings were made with an Axopatch 700B (Molecular Devices), a Digidata 1322A digitizer (Molecular Devices), and Clampex 10 recording software with a sampling rate of 10kHz. Current clamp recordings were adjusted for junction potential (13mV). For all voltage-clamp experiments, holding voltage was V_H_= -60mV, and only neurons with holding currents not exceeding 100pA for the 10-min control period (input resistance > 150 MΩ) were studied further. Blockers were administered for 10min before application of FGF1, which was administered for up to 15min. Neurons were not considered for further analysis if series resistance exceeded 24MΩ or drifted >10% during baseline. All compounds were purchased from Tocris Cookson, Sigma Aldrich and FGF1 from Prospec (Ness Ziona, Israel).

### Intracerebroventricular Cannulation Surgeries

Lateral-ventricle i.c.v. cannulations were performed as previously described (8). In short, animals were anesthetized under 3% isoflurane in oxygen delivered by nose cone. Using a stereotax (David Kopf Instruments, Tujunga, CA), mice were implanted with a cannula (PlasticsOne, Roanoke, VA) in the lateral ventricle: −0.7 mm posterior to bregma, −1.2 mm lateral to the midsagittal suture, and −2.0 mm below the skull surface. Mice recovered for at least 7 days prior to testing. For verification of cannula placement, angiotensin II (2.5 µg/mouse, 5-µL injection volume; Sigma-Aldrich) was administered *via* i.c.v. injection and water intake was recorded over the course of one hour. Mice that drank <1 mL were excluded from the study.

### c-Fos Immunohistochemistry

Mice received a single i.c.v. injection of vehicle (saline) or FGF1 (3µg, dissolved into saline) over 10 s in a final volume of 4 µL using an injector with a 1-mm projection. 90 min later mice were anesthetized with ketamine/xylazine and perfused with 0.9% saline followed by 4% paraformaldehyde. Brains were removed, cryoprotected in 20% sucrose, frozen, and cut at 25 µm in a 1:6 series using a sliding microtome. Sections were stained for c-Fos using a polyclonal rabbit anti–c-Fos antibody (1:10,000, SC-52; Santa Cruz Biotechnology), amplified (PK-4000; Vectastain ABC HRP kit), and visualized with nickel-DAB (DAB Kit, SK-4100; Vector Laboratories). Sections were mounted on slides and imaged with an Olympus brightfield slide scanner. c-Fos immunoreactivity–positive cells were manually counted (blinded) using ImageJ software. Recombinant murine FGF1 was purchased from Prospec (Ness Ziona, Israel) and individual aliquots were stored at -20C.

### Statistical Analysis

Statistical comparison of effect between groups was made using a student t-test or one-way ANOVA with Dunnett’s *post hoc* analysis (unless otherwise noted). For electrophysiology experiments *n* = 4-7 mice was used to control for biological variability between animals, and statistics were performed with total number of cells collected for each experiment. In repeated-measures experiments with missing data points a mixed-model design was used. The Kolmogorov-Smirnov (KS) test was used to determine the significance of drug effect on IPSC frequency within individual neurons (Mini Analysis, Synaptosoft). Membrane potential was calculated by averaging the final 3-min of data collection before switching solutions. In-text qualitative classification of neurons in current-clamp studies used a cut off of a 0.5 mV change, which exceeds the mean of our previously reported vehicle-treated neurons (31). No qualitative classifications were used for statistical analysis. For all experiments, error bars are presented as mean ± standard error of the mean (SEM). Statistics were calculated using Prism7 software (Graphpad).

## Results

### FGF1 Decreases Inhibitory Inputs and Depolarizes ARH-POMC-EGFP

**Figure 1:**
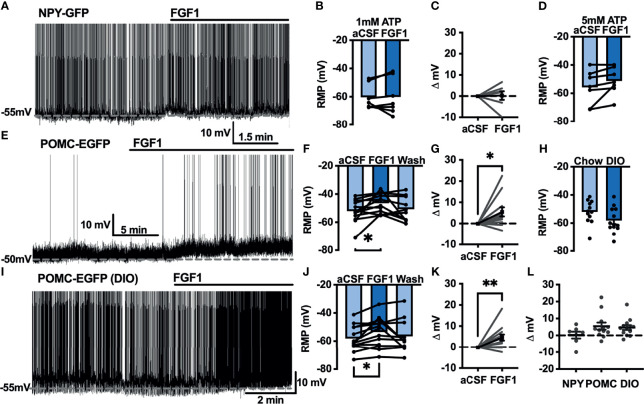
FGF1 depolarizes ARH-POMC-EGFP neurons in chow-fed and DIO mice. **(A)** Representative trace and **(B)** mean membrane potential (RMP; *n* = 7; F_(2, 11)_ = 0.174, *p* = 0.842 *Wash not shown*) from ARH-NPY-GFP neurons before and after bath application of FGF1 (100 nM) with 1mM and **(D)** 5mM internal ATP (*n* = 7; F_(2, 12)_ = 3.20, *p* = 0.077 *Wash not shown*). **(E)** Representative trace and **(F)** mean membrane potential of ARH-POMC-EGFP neurons from lean (*n* = 12; F_(2, 20)_ = 4.25; *p* = 0.029) and **(I, J)** DIO mice (*n* = 13; F_(2, 22)_ = 3.79, *p* = 0.038) before and after bath application of FGF1 (100 nM). **(C)** Distribution plot of mV change in individual neurons from chow-fed NPY-GFP (FGF1: 4.7 ± 1.4 mV; t = 0.128, *p* = 0.902), **(G)** POMC-EGFP (FGF1: 5.5 ± 2.1 mV; t = 2.60, *p* = 0.025), and **(K)** DIO POMC-EGFP (FGF1: 4.5 ± 1.6 mV; t = 3.28, *p* = 0.007) mice. **(H)** Baseline membrane potential in chow vs DIO [t(23) = 1.79, *p* = 0.087] and **(L)** mV change after FGF1 application compared between NPY-GFP, POMC-EGFP, and DIO POMC-EGFP cells. *p < 0.05, **p < 0.01.

Our previous work demonstrated that ICV injection of FGF1 induces c-Fos activation in the ARH, median eminence, and 3^rd^ ventricular tanycytes of control and DIO mice. We hypothesized that FGF1 would alter the activity of ARH-NPY and -POMC neurons (8). To test whether FGF1 alters the activity of ARH-NPY or -POMC neurons whole-cell patch-clamp recordings were performed on ARH-NPY-GFP or ARH-POMC-EGFP neurons. In ARH-NPY-GFP neurons, bath application of FGF1 (100 nM) did not alter the mean membrane potential with a 1mM (**Figures 1A–C, L**) or 5mM (**Figure 1D**) internal ATP concentration. In 10 out of 12 ARH-POMC-EGFP neurons, FGF1 (100 nM) depolarized the membrane potential 2-3min after bath application and was reversible in most cells (**Figures 1E–G, L**). We next recorded from ARH-POMC-EGFP neurons in diet-induced obesity (DIO) mice to determine if this effect is conserved after challenge with a high-fat diet (HFD). In DIO mice, FGF1 depolarized 11 out of 13 ARH-POMC-EGFP neurons (**Figures 1I–K, L**) with no effect of DIO on basal membrane potential (**Figure 1H**).

**Figure 2:**
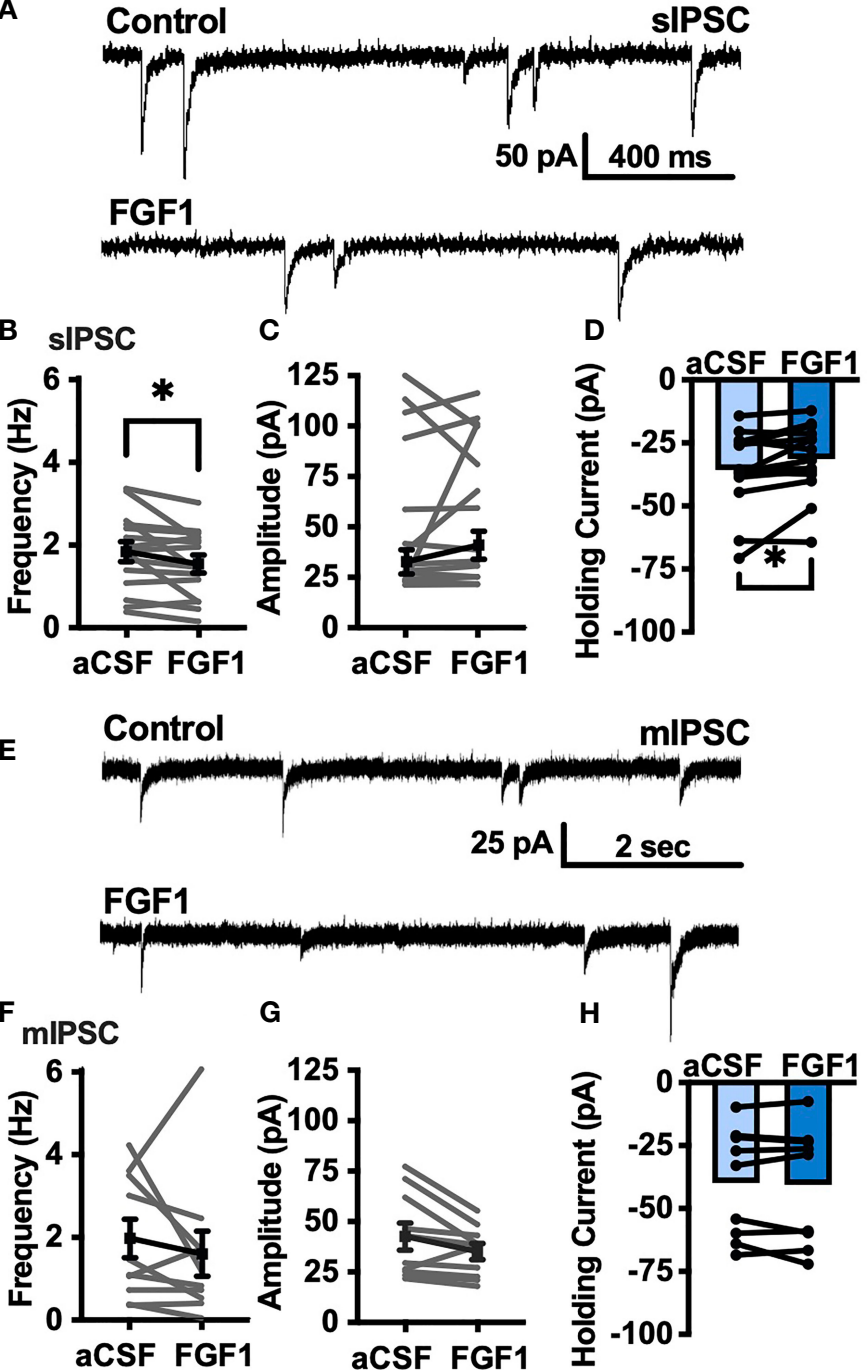
FGF1 decreases sIPSC frequency in ARH-POMC-EGFP neurons. **(A)** Representative trace from a voltage-clamp experiment showing sIPSC frequency and amplitude after bath application of FGF1 (100 nM) in ARH-POMC-EGFP neurons. **(B)** sIPSC mean frequency [*n* = 15, t(14) = 2.37, *p* = 0.033], **(C)** amplitude [t(14) = 1.27, *p* = 0.223] and **(D)** holding current [t(14) = 2.72, *p* = 0.017] before and after bath application of FGF1. **(E)** Representative trace and **(F)** mIPSC mean frequency [*n* = 10; t(9) = 0.814, *p* = 0.437], **(G)** amplitude [t(9) = 2.11, *p* =0.064] and **(H)** holding current [t(9) = 0.574, *p* = 0.582] before and after bath application of FGF1. *p < 0.05.

**Figure 3:**
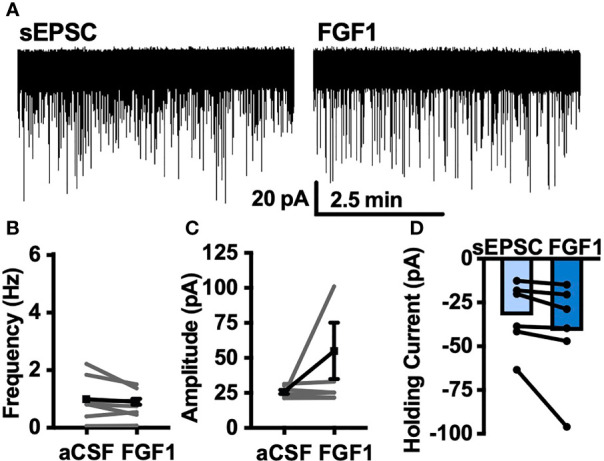
FGF1 does not alter sEPSC frequency or amplitude in ARH-POMC-EGFP neurons. **(A)** Representative trace from a voltage-clamp experiment showing sEPSC frequency and amplitude after bath application of FGF1 (100 nM) in ARH-POMC-EGFP neurons. **(B)** sEPSC mean frequency [*n* = 7, t(6) = 1.57, *p* = 0.167], **(C)** amplitude [t(6) = 149, *p* = 0.186] and **(D)** holding current [t(5) = 1.78, *p* = 0.135] before and after bath application of FGF1.

To determine whether the depolarizing effects of FGF1 on ARH-POMC-EGFP neurons involved a change in inhibitory neurotransmission we measured spontaneous inhibitory postsynaptic currents (sIPSCs) from ARH-POMC-EGFP neurons. Bath application of FGF1 decreased the frequency of sIPSCs in ARH-POMC-EGFP neurons (**Figures 2A, B**), had no effect on amplitude (**Figures 2A, C**), but increased the holding current (**Figures 2A, D**). Additionally, when split into groups of up-responders and down-responders (as determined by a within-cell KS test), 73% of ARH-POMC-EGFP neurons had a significant reduction of sIPSC frequency, in line with the ~80% of ARH-POMC-EGFP neurons that were depolarized in **Figure 1**. To determine if FGF1 impacted excitatory inputs onto ARH-POMC-EGFP neurons we measured sEPSCs and did not observe an effect on frequency or amplitude (**Figures 3A–C**). However, there was a trend in increased holding current which is consistent with the depolarizing effect of FGF1 on ARH-POMC-EGFP neurons (**Figure 3D**).

To determine whether changes in sIPSC frequency were due to direct presynaptic actions or indirect actions occurring upstream, we isolated the synapse and recorded miniature (m) IPSCs by bath applying the voltage-gated sodium channel (VGSC) blocker tetrodotoxin (TTX; 1 μM). FGF1 did not have a significant effect on the frequency of mIPSCs (**Figures 2E, F**) or amplitude (**Figures 2E, G**), However, when split into groups of up-responders and down-responders, 67% of ARH-POMC-EGFP neurons had a reduction in mIPSC frequency, slightly less than the 83% of ARH-POMC-EGFP neurons that were depolarized in **Figure 1**. Interestingly, TTX also blocked FGF1 effects on the holding current of ARH-POMC-EGFP neurons (**Figure 2H**). This suggests that FGF1 acts upstream with an indirect effect on these neurons, and/or VGSCs play a direct role in FGF1 signaling.

### VGSCs and VEGFRs Are Required for FGF1 Activation of ARH-POMC-EGFP Neurons

**Figure 4:**
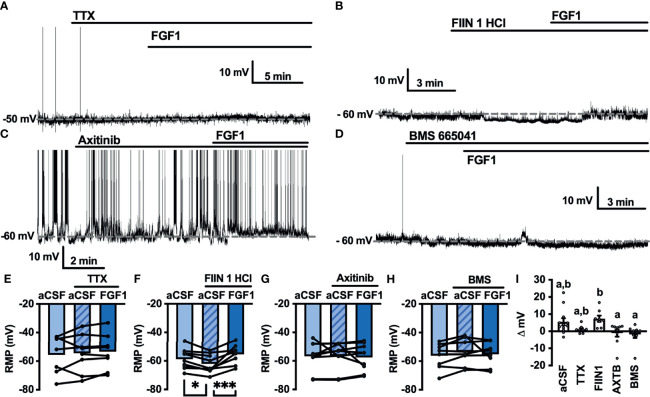
FGF1 depolarizes ARH-POMC-EGFP neurons independent of FGF receptors. **(A)** Representative trace of a POMC-EGFP neuron after bath application of FGF1 in the presence of TTX, **(B)** FIIN 1 HCl **(C)**, axitinib and **(D)**, BMS 665041. **(E)** Mean membrane potential (RMP) after bath application of FGF1 in the presence of TTX (*n* = 8, F_(2, 13)_ = 0.798, *p* = 0.471). **(F)** FIIN HCl (*n* = 8, F_(2, 15)_ = 13.44, *p* < 0.0001), **(G)** axitinib (*n* = 9, F_(2, 16)_ = 0.861, *p* = 0.861) and **(H)** BMS 665041 (*n* = 8, F_(2, 13)_ = 0.798, *p* = 0.471) **(I)** Comparison of mV change after bath application of FGF1 in aCSF (5.5 ± 2.1 mV), TTX (1.1 ± 0.8 mV), FIIN 1 HCl (7.4 ± 1.8 mV), axitinib (-1.0 ± 2.1 mV), and BMS 665041 (-1.7 ± 2.1 mV; F_(4, 40)_ = 4.161, *p* = 0.007). *p < 0.05, ***p < 0.001 (groups not sharing a letter are significantly different from each other as determined by Tukey’s *post hoc*).

Next, we hypothesized that VGSCs may be required for FGF1induced depolarization of ARH-POMC neurons. To test whether VGSCs are necessary for FGF1 mediated depolarization of ARH-POMC neurons we recorded the membrane potential of ARH-POMC-EGFP neurons in the presence of TTX. In this preparation FGF1 did not alter membrane potential in ARH-POMC-EGFP neurons (**Figures 4A, E, I**).

A previous study demonstrated that multiple FGFs bind to the C-terminus of VGSCs (32). In combination with our findings, this led us to question whether FGF receptors (R) are required for FGF1 actions or if its actions on ARH-POMC neurons are mediated by alternative mechanisms. We recorded the membrane potential of ARH-POMC-EGFP neurons in the presence of the irreversible and FGFR 1-4 inhibitor FIIN 1 HCl (FIIN1; 500 nM). Pretreatment with FIIN1 hyperpolarized the neurons, but was not sufficient to block the depolarizing effects of FGF1 (**Figures 4B, F, I**).

It is well documented that VGSCs regulate VEGF signaling and are required for VEGF mediated pERK1/2 activation (33). VEGF-A is expressed in the ARH, mediates plasticity of brain barriers, and blockade of VEGFR decreases ARH leptin sensitivity (34, 35). Given previous findings showing that FGF1 increases pERK1/2 in the ARH, activates tanycytes in the median eminence, which is sensitive to VEGF-A signaling, and our current findings that FGF1 effects on ARH-POMC-EGFP neurons are dependent on VGSCs, but independent of FGFRs, we hypothesized that VEGFRs may be required for FGF1 actions on ARH-POMC neurons (5, 8, 35). We pretreated ARH-POMC-EGFP brain slices with the potent VEGFR 1-3 inhibitor axitinib (100 nM). In the presence of axitinib, FGF1 no longer depolarized ARH-POMC-EGFP neurons (**Figures 4C, G, I**). To confirm this finding, we repeated this experiment with the competitive and selective VEGFR -2, -1 inhibitor BMS 605541 (BMS; 1 μM). As with axitinib, pretreatment with BMS was sufficient to block FGF1 actions on ARH-POMC-EGFP neurons (**Figures 4D, H, I**). These data show that FGF1 activation of ARH-POMC-EGFP neurons is VGSC and VEGFR dependent, likely through the inhibition of presynaptic GABA release or direct actions on ARH-POMC neurons.

### FGF1 Activates Cells in the DVC *In Vivo* and *Ex Vivo*

**Figure 5:**
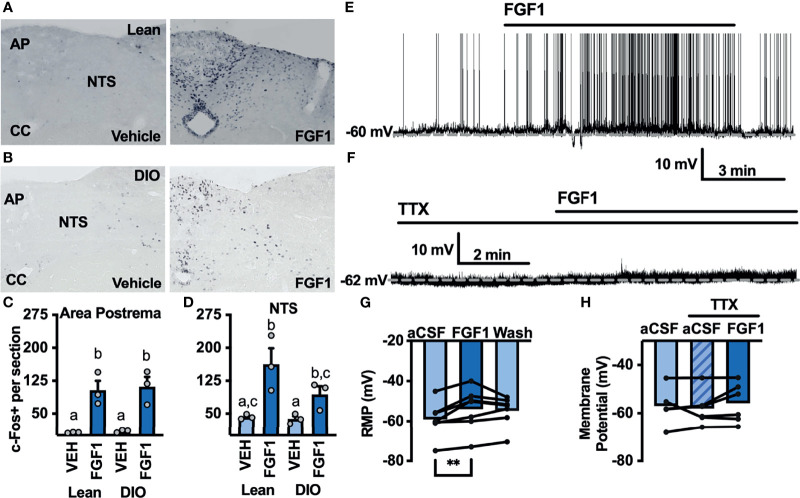
ICV FGF1 injection induces c-Fos in the dorsal DVC **(A)** Representative images of FGF1 induces c-Fos activation in the area postrema and NTS of lean and **(B)** DIO mice **(C)** Mean c-Fos counts (2-3 sections/mouse) in the area postrema (two-way ANOVA, drug effect: F_(1, 9)_ = 47.65, *p* < 0.0001; diet effect: F_(1, 9)_ = 0.140, *p* = 0.717) and **(D)** NTS (drug effect: F_(1, 9)_ = 18.35, *p* = 0.003; diet effect: F_(1, 8)_ = 3.19, *p* = 0.112) of lean (*n* = 3) and DIO (*n* = 3) mice. **(E)** Representative trace and **(F)** mean membrane potential after FGF1 application onto unidentified area postrema neurons (RMP; *n* = 7, F_(2, 12)_ = 7.08, *p* = 0.009) and **(G, H)** after FGF1 application in the presence of TTX (*n* = 7, F_(2, 9)_ = 0.855, *p* = 0.457). (**p < 0.01, groups not sharing a letter are significantly different from each other as determined by Tukey's post hoc).

The DVC plays a pivotal role in mediating peripheral and central metabolic signals, and FGF1 injection into the cisterna magna significantly decreases blood glucose and food intake in *db/db* mice (8). We hypothesized that central administration of FGF1 would activate cells in the DVC. To test this hypothesis we gave an i.c.v injection of FGF1 into the lateral ventricle of lean and DIO cannulated mice and used immunohistochemistry to test for changes in c-Fos expression within the DVC. c-Fos expression was increased throughout the DVC, particularly in the AP and NTS of lean (**Figures 5A, C**) and DIO mice (**Figures 5B, D**). This robust increase in c-Fos expression supports our hypothesis that the DVC plays a role in the metabolic actions of FGF1.

Particularly in the AP, c-Fos activation appeared to be distributed between bot glia and neuron cell types. We hypothesized that FGF1 mediated c-Fos activation in the AP would translate into direct actions of FGF1 on AP neurons. To investigate the actions of FGF1 on neurons in the AP we recorded the membrane potential of unidentified AP neurons and found that FGF1 ubiquitously depolarized these neurons (**Figures 5E, G**). Similar to ARH-POMC neurons, this effect was blocked in the presence of TTX, suggesting that a similar mechanism involving VGSCs may mediate the effects of FGF1 within the AP (**Figures 5F, H**).

**Figure 6:**
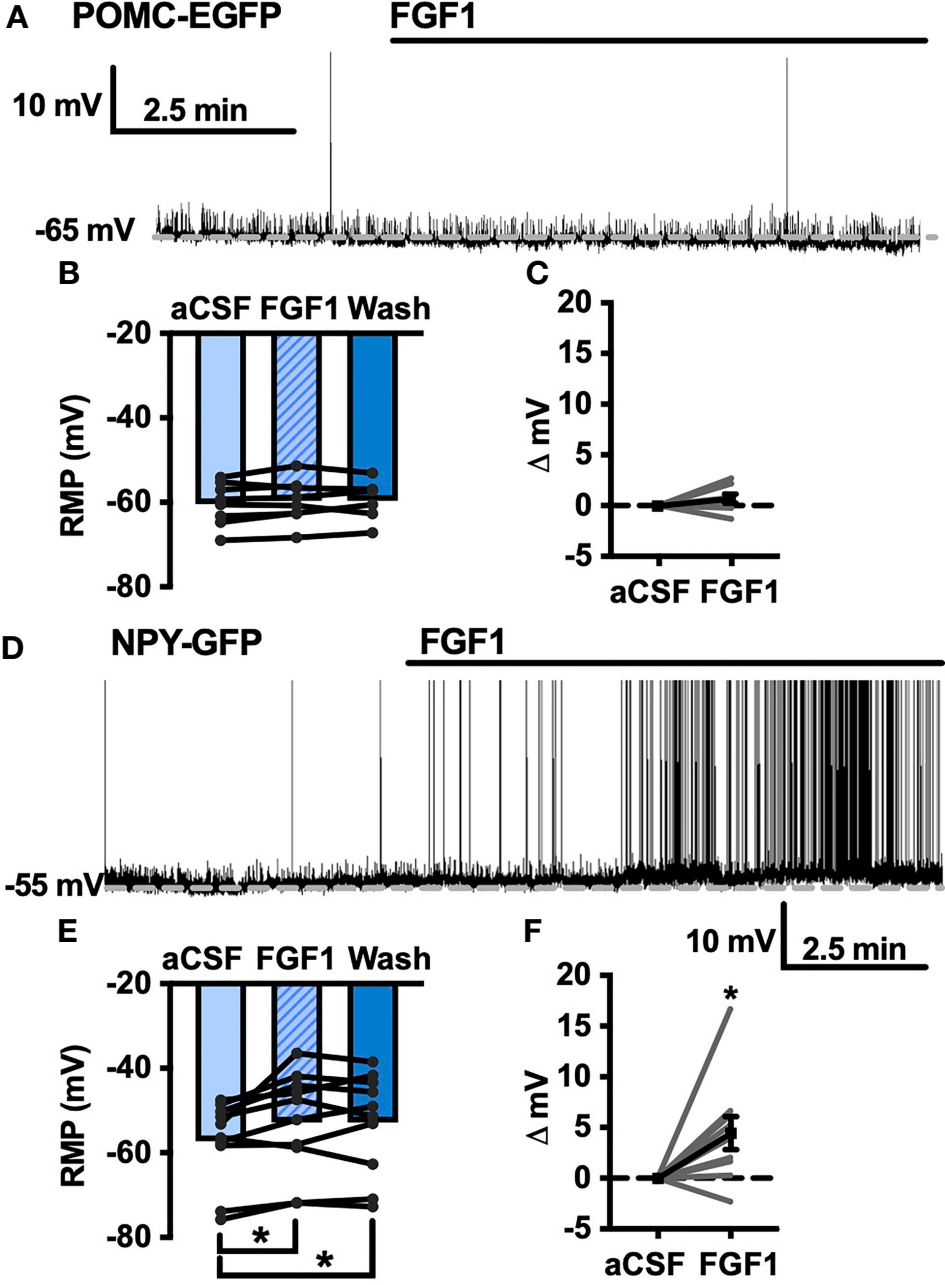
FGF1 activates NTS-NPY-GFP neurons. **(A)** Representative trace, **(B)** mean membrane potential (RMP; *n* = 7, F_(2, 12)_ = 1.05, *p* = 0.379), and **(C)** Plot of net mV change in NTS-POMC-EGFP neurons before and after bath application of FGF1 [0.73 ± 1.3 mV; t(8) = 1.63, *p* = 0.147]. **(D)** Representative trace and **(E)** mean membrane potential change for NTS-NPY-GFP neurons (*n* = 10, F_(2, 12)_ = 5.91, *p* = 0.011). **(F)** Plot of net mV change in NTS-NTS-GFP neurons before and after bath application of FGF1 [4.5 ± 1.6 mV; t(9) = 2.75, *p* = 0.022]. *p < 0.05.

To further our investigation into the actions of FGF1 in the DVC we transitioned to the NTS, which serves as a relay center between the DVC and rest of the brain, including the ARH (10–12). We hypothesized that FGF1 c-Fos activation in the NTS would translate to direct physiological activation of NTS neurons. Given the actions of FGF1 on ARH-POMC-EGFP neurons, we first tested this hypothesis by recording from NTS-POMC-EGFP neurons. Interestingly, bath application of FGF1 on NTS-POMC-EGFP neurons did not affect membrane potential (**Figures 6A–C**). Next we recorded from NTS-NPY-GFP neurons, which play a known role in glucoprivic feeding behavior and unlike ARH-NPY neurons, colocalize with noradrenergic catecholamine neurons in the NTS, as well as other hindbrain regions implicated in energy homeostasis (36). Unlike ARH-NPY-GFP neurons, bath application of FGF1 depolarized the membrane potential of NTS-NPY-EGFP neurons (**Figures 6D–F**).

### FGF1 Actions on NTS-NPY-GFP Neurons Involves FGF and VEGF Receptors

**Figure 7:**
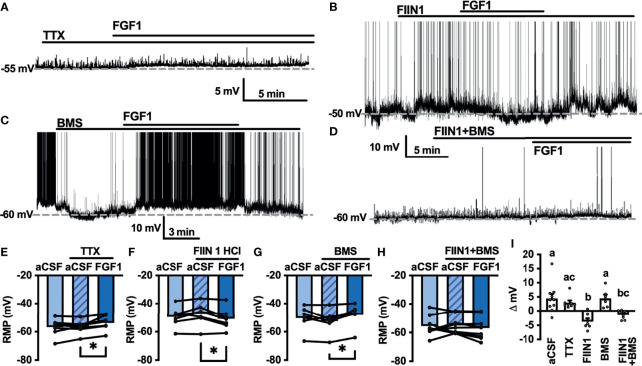
FGF1 acts on NTS-NPY-GFP neurons through multiple mechanisms. **(A)** Representative trace and **(E)** mean membrane potential (RMP) before and after bath application of FGF1 in the presence of TTX (*n* = 8, F_(2, 13)_ = 5.37, *p* = 0.020) **(B, F)** FIIN 1 HCl (*n* = 7, F_(2,12)_ = 4.86, *p* = 0.029) **(C, G)** BMS (*n* = 6, F_(2, 10)_ = 6.11, *p* = 0.019) and **(D, H)** FIIN1 + BMS (*n* = 9, F_(2, 16)_ = 0.934, *p* = 0.413). **(I)** Comparison of mV change after bath application of FGF1 (F_(4, 33)_ = 8.28, *p* < 0.0001) in aCSF (4.5 ± 1.6 mV), TTX (3.0 ± 0.9 mV), FIIN1 (-3.7 ± 1.2 mV), BMS (4.5 ± 1.3 mV), and FIIN + BMS (-1.3 ± 0.5 mV). *p < 0.05 (groups not sharing letters represent a significant difference of *p* < 0.05 using Tukey’s *post hoc*].

As shown in **Figures 2**,**4** and **5**, FGF1 activation of ARH-POMC-EGFP and unidentified AP neurons requires VGSCs, as the presence of TTX blocked the effects of FGF1. We hypothesized that FGF1 actions on NTS-NPY-GFP neurons would involve a similar mechanism. To test this, we measured the membrane potential of NTS-NPY-GFP neurons in the presence of TTX and bath applied FGF1. Interestingly, TTX was not sufficient to block the effects of FGF1 on membrane potential in these neurons (**Figures 7A, E, I**). This suggests that FGF1 is acting directly on NTS-NPY-GFP neurons and through a separate or additional mechanism compared to ARH-POMC-EGFP and AP neurons.

In ARH-POMC-EGFP neurons, the FGFR inhibitor FIIN1 was not sufficient to block the effects of FGF1 (**Figures 4B, F, I**). However, previous work shows that FGFR expression, particularly FGFR-1, is much higher in the NTS than the ARH or AP (37). To determine whether FGFRs are required for FGF1 activation of NTS-NPY-GFP neurons we bath applied FGF1 in the presence of FIIN1, which revealed a hyperpolarizing effect of FGF1 on the membrane potential, opposite of our observations when applying FGF1 alone (**Figures 7B, F, I**). This result led us to hypothesize that FGF1 may depolarize NTS-NPY-GFP neurons through FGFRs, which provide an inhibitory tone on NTS-NPY neurons through an additional unknown mechanism. Given that FGF1 actions on ARH-POMC-EGFP neurons requires VEGFRs, we hypothesized that VEGFRs may play a role in FGF1 signaling onto NTS-NPY-GFP neurons. When pretreating with the VEGFR -2, -1 inhibitor BMS, FGF1 actions on NTS-NPY-GFP neurons was not altered (**Figures 7C, G, I**). However, the combination of both FIIN1 and BMS was sufficient to block FGF1 effects on the membrane potential of NTS-NPY-GFP neurons, suggesting that FGFRs and VEGFRs have opposing roles in FGF1 signaling onto NTS-NPY-GFP neurons (**Figures 7D, H, I**).

## Discussion

The FGF family plays a well-documented role in metabolic processes; however, little is known regarding the electrophysiological mechanisms underlying the actions of FGF1 in brain regions involved with energy homeostasis. Recent literature has demonstrated that, in addition to inducing the remission of diabetes in rodent models, FGF1 induces robust activation of c-Fos and pERK1/2 in the ARH and median eminence (ME), however, the neuronal phenotypes and extent of neuronal activation in the ARH are less clear (5, 7). Additionally, much of the literature has focused on the long-term effects of FGF1 using diabetic rodent models with disrupted leptin signaling, and much less is known about the mechanisms underlying acute effects of FGF1, which are independent of long-term reductions in hyperglycemia (5, 8, 20). Here we demonstrate that FGF1 does not acutely modulate the activity of orexigenic ARH-NPY neurons. However, it should be considered that we used a single concentration of FGF1 with a low time course compared to *in vivo* studies. This result supports studies demonstrating that FGF1 increases pERK1/2 in tanycytes and glia, but not neurons (5). In contrast, our results show FGF1 activates the anorexigenic ARH-POMC neurons in both chow-fed and DIO mice. The effect of FGF1 was partially reversible in most cells suggesting it is unlikely that the acute electrophysiological response of ARH-POMC neurons alone is responsible for sustained improvements in hyperglycemia. The anorexigenic effect of ARH-POMC neuronal activation has a slow onset and acts, in part, by increasing synaptic plasticity in the paraventricular nucleus of the hypothalamus (PVH) through the postsynaptic release of α-MSH onto PVH neurons (38–40). The results here promote further investigation into whether downstream signaling cascades, such as plasticity in the ARH-POMC→PVH pathway, contributes to the prolonged anorexigenic effects of FGF1, and how the kinetics of FGF1 actions differ in the *ob*/*ob* and *db*/*db* models of hyperglycemia used in previous studies.

ARH-POMC neurons receive inhibitory GABAergic inputs from multiple brain regions, including ARH-NPY neurons (17). Here we show that in the majority of ARH-POMC neurons, FGF1 reduces the frequency of inhibitory inputs, which may contribute to the excitatory effects of FGF1. To determine if FGF1 decreases the frequency of inhibitory inputs directly or by modulating upstream neurons, we isolated the synapse by inhibiting action potential firing with the VGSC blocker TTX and observed a decrease in mIPSC frequency in a subset of ARH-POMC neurons. In addition to presynaptic GABA release, FGF1 actions on ARH-POMC neurons may be mediated by activation of VGSCs as TTX blocked FGF1-mediated changes in holding current. Although these experiments are limited by low sample size, this result is supported by our data showing TTX alone is sufficient to block the depolarizing effects of FGF1 and suggests that FGF1 may act through a non-FGFR mediated mechanism in the ARH.

Recent work by Hultman et al. provides a comprehensive map of FGFR expression throughout the mouse and non-human primate brain (37). Of interest, this work shows very low expression levels of all FGFRs in the ME, and very low expression of FGFR-1, -2 and -4 in the ARH and majority of α-tanycytes. Yet, a combination of the literature and work presented here clearly demonstrate that FGF1 induces cellular activation at all these sites. We used the FGFR inhibitor FIIN1 to determine whether FGFRs are required for FGF1 to depolarize ARH-POMC neurons and show that FGF1 depolarizes ARH-POMC neurons independent of FGFRs.

With the observation that VGSCs are required, but not FGFRs, for FGF1 actions on ARH-POMC neurons, we investigated the possible role of VEGFRs, which are known to interact with VGSCs and play a metabolic role in the hypothalamus (33, 35). Surprisingly, both the general VEGFR inhibitor axitinib and VEGFR -1, -2 inhibitor BMS blocked FGF1 effects on ARH-POMC neurons. While the combination of these results does not rule out the role of FGFRs in ARH-POMC neurons, they do demonstrate that FGF1 acts through multiple mechanistic pathways and that VGSCs and VEGFRs mediate FGF1 depolarization of ARH-POMC neurons.

In addition to the hypothalamus, the hindbrain plays a major role in energy homeostasis. In particular, the DVC is an important interface for relaying information between the brain and periphery (10–12, 23). It is well documented that neurons within the DVC play a vital role in controlling blood glucose, glucoprivic and lipoprivic feeding responses (22, 23, 28, 41). We investigated the actions of FGF1 in two regions of the DVC, the AP and NTS, and observed increased c-Fos in both regions. To determine whether this effect was conserved *ex vivo*, we recorded the electrophysiological actions of FGF1 on AP neurons and found that FGF1 depolarized these neurons in a VGSC dependent manner, similar to our findings in ARH-POMC neurons.

Next, we transitioned to the NTS to investigate whether FGF1 had actions on NTS-POMC neurons. NTS-POMC neurons receive direct inputs from vagal afferent fibers, respond to regulators of appetite, and activation of these neurons results in immediate inhibition of feeding behavior (40, 42). Here we saw no effect of FGF1 on NTS-POMC neurons and future studies are needed to determine whether they have any involvement in FGF1 signaling. The NTS contains A2/C2 catecholamine neurons and the majority of noradrenergic C2 cell bodies, as well as other hindbrain catecholamine neurons, co-express NPY (36, 43). Our results show that FGF1 is sufficient to depolarize NTS-NPY neurons. NPY neurons in the hindbrain play a substantial role in the regulation of glucoprivic feeding, which is mediated in part by the innervation of NPY fibers into the PVH (36, 43–46). Counter to our findings, recent work demonstrated that activation of NTS-NPY neurons stimulates feeding (47). In this recent study the orexigenic NTS-NPY neurons were colocalized with the more rostral and medial adrenergic neurons, whereas our recordings were performed in the caudal/medial A2 region and may be anatomically distinct (36, 43, 47). Additionally, others have shown that anorexigenic compounds, such as nicotine, are sufficient to activate NTS-NPY neurons (48).

To further understand the mechanism by which FGF1 acts on NTS-NPY neurons we used TTX to determine if, similar to AP and ARH-POMC neurons, VGSCs were required for FGF1 actions on NTS-NPY neurons. Surprisingly, administration of TTX was not sufficient to block the effects of FGF1 in these neurons. Expression of FGFRs, particularly FGFR-1, in the NTS is higher than in the AP, ARH, and surrounding regions (37). This led us to test whether FGFRs mediate the effects of FGF1 in NTS-NPY neurons. Here we show that the FGFR 1-4 inhibitor FIIN1 did not inhibit FGF1 actions, but revealed a hyperpolarizing effect of FGF1 in NTS-NPY neurons. This suggested that FGF1 is acting through multiple mechanisms in NTS-NPY neurons, an excitatory effect mediated by FGFRs and an unknown inhibitory component. Surprisingly, FINN1 alone depolarized NTS-NPY neurons and future work is needed to determine whether there is tonic FGFR activity in this region. To determine whether VEGFRs contributed to this inhibitory component we co-applied FIIN1 with the VEGFR -1, -2 inhibitor BMS. This combination was sufficient to completely block FGF1 actions on these neurons, suggesting that FGFRs and VEGFRs play opposing mechanistic roles in NTS-NPY neurons. This also implies that VEGFR mediated effects of FGF1 are opposite in ARH-POMC and NTS-NPY neurons, reminiscent of the actions of other anorexigenic proteins, such as leptin, on ARH-POMC and ARH-NPY neurons (49). Whether either of these mechanisms is disrupted in diabetic rodent models or if *in vivo* FGF1 administration alters membrane receptor expression is yet to be determined and a question of future interest.

In summary, here we present the groundwork for understanding the mechanistic actions of FGF1 in the ARH and DVC. We demonstrate that FGF1 depolarizes ARH-POMC, NTS-NPY, and unidentified AP neurons. Second, FGF1 actions on ARH-POMC and AP neurons require VGSCs. Lastly, we demonstrate that FGF1 actions in ARH-POMC and NTS-NPY neurons are mediated, in part, by VEGFRs.

The work presented here addresses the acute actions of FGF1, but is only a starting point in understanding the scope of how FGF1 signaling impacts neurophysiological properties in brain regions. Given that the effects of FGF1 can last for weeks after treatment, future studies are required to address whether FGF1 induces synaptic remodeling of neurons in the hypothalamus or DVC. Myriad studies have characterized the long-term effects of FGF1 on hyperglycemia and food intake, with differing results depending on the animal model and route of administration (5–8). It is unknown whether the mechanisms described here are conserved in DIO or diabetic rodent models and future studies are necessarily to fully understand these differences. This study does open new paths for the future investigation of central mechanisms mediating the effects of FGF1 on food intake and blood glucose levels. To the best of our knowledge, this is the first study highlighting the role of FGF1 in hindbrain signaling and demonstrating that FGF1 has mechanistic actions independent of FGFRs.

## Data Availability Statement

The original contributions presented in the study are included in the article/supplementary material. Further inquiries can be directed to the corresponding author.

## Ethics Statement

The animal study was reviewed and approved by the Oregon National Primate Research Center Animal Institutional Care and Use Committee.

## Author Contributions

All authors contributed to study design, data collection, analysis and interpretation, or drafting and revising of manuscript, and approved the final manuscript for publication. Specifically, BLR and PK designed the study. BLR, KGT, EJK and SRL contributed to data collection. BLR and EJK contributed to data analysis. BLR and PK contributed to data interpretation, and preparation of the manuscript.

## Funding

This work was supported in part by NIH grant P51 OD 01192 for operation of the Oregon National Primate Research Center.

## Conflict of Interest

The authors declare that the research was conducted in the absence of any commercial or financial relationships that could be construed as a potential conflict of interest.

## Publisher’s Note

All claims expressed in this article are solely those of the authors and do not necessarily represent those of their affiliated organizations, or those of the publisher, the editors and the reviewers. Any product that may be evaluated in this article, or claim that may be made by its manufacturer, is not guaranteed or endorsed by the publisher.
